# Updated recommendations: an assessment of NICE clinical guidelines

**DOI:** 10.1186/1748-5908-9-72

**Published:** 2014-06-11

**Authors:** Laura Martínez García, Emma McFarlane, Steven Barnes, Andrea Juliana Sanabria, Pablo Alonso-Coello, Philip Alderson

**Affiliations:** 1 Institute of Biomedical Research (IIB Sant Pau), Barcelona, Iberoamerican Cochrane Centre, Hospital de la Santa Creu i Sant Pau, Pavelló 18 C/Sant Antoni Mª Claret 167, Barcelona, Catalunya 08025, España; 2 National Institute for Health and Care Excellence, Manchester, UK

**Keywords:** Clinical practice guidelines, Information dissemination, Evidence-based medicine, Knowledge translation, Methods, Updating

## Abstract

**Background:**

Updating is important to ensure clinical guideline (CG) recommendations remain valid. However, little research has been undertaken in this field. We assessed CGs produced by the National Institute for Health and Care Excellence (NICE) to identify and describe updated recommendations and to investigate potential factors associated with updating. Also, we evaluated the reporting and presentation of recommendation changes.

**Methods:**

We performed a descriptive analysis of original and updated CGs and recommendations, and an assessment of presentation formats and methods for recording information. We conducted a case-control study, defining cases as original recommendations that were updated (‘new-replaced’ recommendations), and controls as original recommendations that were considered to remain valid (‘not changed’ recommendations). We performed a comparison of main characteristics between cases and controls, and we planned a multiple regression analysis to identify potential predictive factors for updating.

**Results:**

We included nine updated CGs (1,306 recommendations) and their corresponding original versions (1,106 recommendations). Updated CGs included 812 (62%) recommendations ‘not reviewed’, 368 (28.1%) ‘new’ recommendations, 104 (7.9%) ‘amended’ recommendations, and 25 (1.9%) recommendations reviewed but unchanged. The presentation formats used to indicate the changes in recommendations varied widely across CGs. Changes in ‘amended’, ‘deleted’, and ‘new-replaced’ recommendations (n = 296) were reported infrequently, mostly in appendices. These changes were recorded in 167 (56.4%) recommendations; and were explained in 81 (27.4%) recommendations. We retrieved a total of 7.1% (n = 78) case recommendations (‘new-replaced’) and 2.4% (n = 27) control recommendations (‘not changed’) in original CGs. The updates were mainly from ‘Fertility CG’, about ‘gynaecology, pregnancy and birth’ topic, and ‘treatment’ or ‘prevention’ purposes. We did not perform the multiple regression analysis as originally planned due to the small sample of recommendations retrieved.

**Conclusion:**

Our study is the first to describe and assess updated CGs and recommendations from a national guideline program. Our results highlight the pressing need to standardise the reporting and presentation of updated recommendations and the research gap about the optimal way to present updates to guideline users. Furthermore, there is a need to investigate updating predictive factors.

## Background

Clinical Guidelines (CGs) are ‘statements that include recommendations intended to optimize patient care, that are informed by systematic reviews of evidence and an assessment of the benefits and harms of alternative care options’ [1]. Scientific knowledge is in constant change, and new information requires frequent assessment to determine whether it changes clinical practice. Therefore, CGs require a periodic review of new scientific research that may change the influencing factors in the formulation of their recommendations (quality of the evidence, balance between benefits and harms, patients’ values and preferences, or resource use and cost [2]). Any decision to update a guideline must balance the need to reflect changes in the evidence against the need for stability, because frequent changes to guideline recommendations would make implementation difficult [3-6]. Furthermore, a requirement to maintain a clinically relevant library of guidelines and the resource use this requires may also be a deciding factor.

Updating CGs is a complex process and includes three main stages: identifying important new evidence; assessing if the new evidence has an impact on the current guideline recommendations and whether an update is required; and the updating process [7]. Some research has been published about the identification and assessment of new evidence (encompassed sometimes as review, surveillance or monitoring process), [8,9]. Less attention has been paid to the process of updating CGs *per se*, making the assumption that it is similar to the development process [7,8].

The role of the National Institute for Health and Care Excellence (NICE) is to improve outcomes for people using the National Health Service in England and Wales and other public health and social care services. Since 2005, NICE has developed CGs which are systematically-developed statements to assist professional and patient decisions about appropriate care for specific clinical circumstances. As advances in medicines and technologies may lead to guideline recommendations becoming obsolete, CGs developed by NICE are published with the expectation that they will be regularly reviewed and updated as necessary [3].

These processes have evolved in different versions of NICE manuals [3-5]. At present, NICE has suspended the routine review process every three years and an interim surveillance programme is being implemented, which alternates rapid (two, six, and ten years after publication) or full reviews (four and eight years after publication) [6]. Also, rapid updates of guidelines are being piloted [10].

Although the updating process is not yet standardised, some institutions try to keep their CG program up to date [11]. An analysis of current practice would provide relevant information about CG review and update process, and also highlight existing gaps in the process. For example, the identification of recommendations with high or low turnover or clinical questions with a greater proportion of updated recommendations will help to focus evidence surveillance systems and, consequently, will improve the distribution of resources in the CG updating process.

Therefore, we evaluated updated CGs from NICE to identify and describe updated recommendations and to investigate factors associated with updating (predictive factors). Also, we assessed presentation formats and methods for recording information when presenting the changes from the original to updated recommendations.

## Methods

### Study design

We performed a descriptive study of original and updated CGs and recommendations. Also, we conducted a case-control study to identify potential predictive factors for updating.

### Setting and participants

We included all updated CGs from NICE; and their corresponding original version. We included an updated CG if: it was partially updated; it was the first updated version of the original; it included the updating status of each recommendation; and it was published on the NICE website up to May 2013. Updated CGs were obtained from the NICE website in May 2013 by searching the list of published CGs ( http://guidance.nice.org.uk/CG/Published). After we obtained the sample of the updated CGs, we retrieved the originals from internal sources within NICE as the original versions of the CGs are no longer available on the website.

Finally we reviewed CGs and selected the recommendations. We included all the recommendations from both original and updated CGs, except research recommendations because these are unanswered research questions or areas of uncertainty that emerge during guidance development as opposed to recommendations for clinical practice.

We defined cases as original recommendations that were updated (‘new-replaced’ recommendations), and controls as original recommendations that were still valid after an updating process (‘not changed’ recommendations).

### Variables and data sources

We mapped original and updated CGs and extracted the following metrics: publication date, topic area (using NICE taxonomy), and guideline development centre (centres that are contracted to develop the guidelines on behalf of NICE). We also extracted from the updated CGs the information about update status labels and definitions (Table 1). We mapped the corresponding original recommendations and extracted: recommendations, heading and subheading, and their strength (with SIGN or GRADE system [2,12]). We coded the recommendations by: CG topic area, years between versions, purpose (using key words), and strength [Additional file 1].

**Table 1:** Update status labels and definitions

Update status label*			Definition*	**Recommendations included in case-control study**
Reviewed	New	New-replaced	Recommendation from original guideline that has been changed following review of evidence.	Cases
		New-added	New recommendation following review of evidence.	
	Not changed		Recommendation from the original guideline where evidence has been reviewed but the recommendation is not changed.	Controls
	Deleted		Recommendation from original guideline that has been removed.	
Not reviewed			Recommendation from the original guideline where the evidence has not been formally reviewed for the update.	
Amended			A small amendment has been made to the original recommendation but the evidence has not been updated or reviewed.	

*Adapted from NICE manual [5].

We mapped and recoded the updated recommendations and extracted similar information plus: update status (Table 1); change recorded (whether changes in the recommendations were registered) [Additional file 1] and justification of the change. For example, the recommendation in Fertility CG 2004 [13], ‘Women who are undergoing *in vitro* fertilisation treatment using gonadotrophin-releasing hormone agonists for pituitary down regulation should be informed that luteal support using human chorionic gonadotrophin or progesterone improves pregnancy rates’; was replaced in Fertility CG 2013 [14] with ‘Offer women progesterone for luteal phase support after *in vitro* fertilisation treatment’; the change was recorded in the guideline as ‘new 2013′ label; and the change was justified as follows ‘New evidence shows that only progesterone is useful as a luteal phase support, so the recommendation has been changed’.

We matched original and updated recommendations; firstly using the changes recorded in the updated CGs, and secondly matched recommendation with recommendation, to obtain the updating status. One reviewer mapped, extracted, and recoded the data. A second evaluator checked the coding and the link processes.

### Data analysis

We performed a descriptive analysis of included CGs and recommendations and calculated absolute and relative frequencies (purpose of recommendations and update status) or median and range (number of recommendations per CG and time between versions of original and updated CGs), as appropriate. We also conducted a descriptive analysis of the update status of recommendations, changes recorded and justification for the change. We assessed the reported information about the change in recommendations per CG based on seven issues (recording information score): recommendation update status defined; changes recorded for ‘amended’ recommendations; changes explained for ‘amended’ recommendations; changes recorded for ‘deleted’ recommendations; changes explained for ‘deleted’ recommendations; changes recorded for ‘new-replaced’ recommendations; and changes explained for ‘new-replaced’ recommendations. For each of the seven issues the selection was one (yes) or zero (no), and the summation was the numerator. The maximum points score were seven (denominator), but depended on the type of update status included in CGs (for example, lung cancer CG 2011 [15] did not have ‘amended’ or ‘new’ recommendations so the denominator was three). The final score was reported on a scale of ten (sum points obtained/highest score possible * 10 = final score).

We compared cases (‘new-replaced’) and controls (‘not changed’) recommendations using Pearson chi-square test (for categorical variables) or Mann-Whitney U test (for quantitative variables), as appropriate. We planned to perform a multiple regression analysis with variables associated with updating in bivariate analysis and with relevant variables agreed by the research group [16]. In addition, we aimed to link recommendations with references supporting them with a view to exploring the association between number of references linked per recommendation and its vulnerability to change.

We accepted p value ≤0.05 as significant in all calculations. We performed the analysis with SPSS 15.0 (SPSS Inc., Chicago, Illinois, United States).

## Results

### Included CGs and recommendations

**Figure 1:**
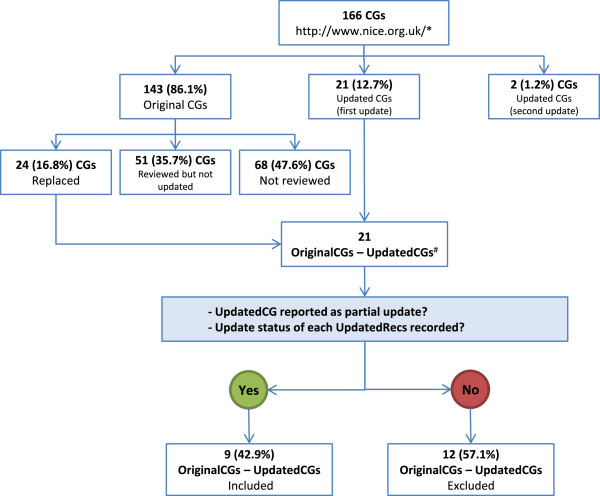
**Flow diagram of included clinical guidelines.** Abbreviations: CG: Clinical guideline. *Accessed 15 May, 2013. ^#^Four OriginalCGs about Type 2 diabetes were updated in one UpdatedCG.

We retrieved 21 (12.7%) updated CGs out of a total of 166 current CGs (Figure 1); and linked them with 24 original CGs (three CGs about type 2 diabetes were merged into one during an update). We excluded 12 updated CGs: eight were not reported as partial update, and four did not record the update status of each recommendation (Additional file 2). Finally, we included nine updated CGs and their corresponding original CGs (Table 2) [13-15,17-31]. Of the study sample, original CGs were published from 2003 to 2006; and updated CGs were published from 2007 to 2013 [Table 2]. The median time between versions was 7.2 years (range 4.3 – 9.0).

**Table 2:** Included clinical guidelines

**Original clinical guidelines**			**Updated clinical guidelines**		
**Title**	**Development centre**	**Publication date**	**Title**	**Development centre**	**Publication date**
- Anaemia management in chronic kidney disease (CG39) (replaced by CG114) [24]	NCC-CC	Sep-06	- Anaemia management in people with chronic kidney disease (CG114) [28]	NCGC	Feb-11
- Caesarean section (CG13) (replaced by CG132) [21]	NCC-WCH	Apr-04	- Caesarean section (CG132) [29]	NCC-WCH	Nov-11
- Chronic heart failure (CG5) (replaced by CG108) [19]	NCC-CC	Jul-03	- Chronic heart failure (CG108) [27]	NCGC-ACC	Aug-10
- Chronic obstructive pulmonary disease (CG12) (replaced by CG101) [20]	NCC-CC	Feb-04	- Chronic obstructive pulmonary disease (updated) (CG101) [26]	NCGC-ACC	Jun-10
- Epilepsy (CG20) (replaced by CG137) [22]	NCC-PC	Oct-04	- Epilepsy (CG137) [30]	NCGC-ACC	Jan-12
- Fertility (CG11) (replaced by CG156) [13]	NCC-WCH	Feb-04	- Fertility (CG156) [14]	NCC-WCH	Feb-13
- Head injury (CG4) (replaced by CG56) (withdrawn) [18]	NCC-AC	Jun-03	- Head injury (CG56) [25]	NCC-AC	Sep-07
- Infection control (CG2) (replaced by CG139) [17]	National Collaborating Centre for Nursing & Supportive Care and Thames Valley University	Jun-03	- Infection control (CG139) [31]	NCGC-ACC	Mar-12
- Lung cancer (CG24) (replaced by CG121) [23]	NCC-AC	Feb-05	- Lung cancer (CG121) [15]	NCC-C	Apr-11

*Abbreviations: NCC-AC* National Collaborating Centre for Acute Care, *NCC-C* National Collaborating Centre for Cancer, *NCC-CC* National Collaborating Centre for Chronic Conditions, *NCC-PC* National Collaborating Centre for Primary Care, *NCC-WCH* National Collaborating Centre for Women and Children’s Health, *NCGC* National Clinical Guideline Centre, *NCGC-ACC* National Clinical Guideline Centre for Acute and Chronic Conditions.

Original CGs included a median of 99 recommendations per CG (range 50 – 220), and 126 (range 52 – 285) for updated CGs (Table 3). The majority of recommendations across all CGs were about ‘gynaecology, pregnancy and birth’ (24.9% of original recommendations and 25.7% of updated recommendations) or ‘respiratory’ topics (25.9% of original recommendations and 23.8% of updated recommendations); were most commonly related to ‘treatment’ purpose or ‘supporting patients or carers’ (27.9% and 19.2% of original recommendations and 28.4% and 18.3% of updated recommendations respectively); and most were graded as ‘D’ or as ‘weak’ recommendations (39.8% of original recommendations and 42.7% of updated recommendations) (Table 3). The references from recommendations to evidence could not be retrieved.

**Table 3:** Recommendations characteristics

	**Original recommendations**		**Updated recommendations**	
	**(n = 1106)**		**(n = 1309)**	
**CGs, n (%)**				
- Anaemia management in chronic kidney disease	50	(4.5)	52	(4.0)
- Caesarean section	108	(9.8)	124	(9.5)
- Chronic heart failure	92	(8.3)	95	(7.3)
- Chronic obstructive pulmonary disease	193	(17.5)	182	(13.9)
- Epilepsy	220	(19.9)	285	(21.8)
- Fertility	167	(15.1)	213	(16.3)
- Head injury	83	(7.5)	126	(9.6)
- Infection control	99	(9.0)	102	(7.8)
- Lung cancer	94	(8.5)	130	(9.9)
**CGs topic, n (%)**				
- Blood and immune system/Urogenital	50	(4.5)	52	(4.0)
- Cardiovascular	92	(8.3)	95	(7.3)
- Central nervous system	220	(19.9)	285	(21.8)
- Gynaecology, pregnancy and birth	275	(24.9)	337	(25.7)
- Injuries, accidents and wounds	83	(7.5)	126	(9.6)
- Public health	99	(9.0)	102	(7.8)
- Respiratory	287	(25.9)	312	(23.8)
**Recommendations purpose, n (%)**				
- Access to services - Referral and approach to care - Service organisation	168	(15.2)	226	(17.3)
- Diagnostic	188	(17.0)	223	(17.0)
- Monitoring - Follow up	64	(5.8)	68	(5.2)
- Prevention practices	153	(13.8)	164	(12.5)
- Supporting patients and carers	212	(19.2)	239	(18.3)
- Treatment	309	(27.9)	372	(28.4)
- >1 purpose or others	12	(1.1)	17	(1.3)
**Recommendations strength - SIGN, n (%)**				
- A	194	(17.5)	133	(14.1)
- B	98	(8.9)	87	(9.2)
- C	140	(12.7)	118	(12.5)
- D	440	(39.8)	402	(42.7)
- GPP	214	(19.3)	187	(19.9)
- Others	20	(1.8)	14	(1.5)
**Recommendations strength - GRADE, n (%)**				
- Legal	-	-	11	(3.0)
- Strong	-	-	49	(13.3)
- Weak	-	-	308	(83.7)
**Update status, n (%)**				
- Amended	94	(8.5)	104	(7.9)
- Deleted	124	(11.2)	-	-
- New – added	-	-	294	(22.5)
- New – replaced	78	(7.1)	74	(5.7)
- Not changed	27	(2.4)	25	(1.9)
- Not reviewed	783	(70.8)	812	(62.0)

*Abbreviations: CG* Clinical guidelines, *GPP* Good practice point.

### Recommendations updating status

Updated CGs included 812 (62%) recommendations ‘not reviewed’; 368 (28.1%) ‘new’ recommendations (‘new-added’ and ‘new-replaced’); 104 (7.9%) ‘amended’ recommendations; and 25 (1.9%) recommendations that were reviewed but remained unchanged (Table 3).

‘Reviewed’ recommendations (‘new’ plus ‘not changed’; 393, 30%) were included in 45 sections of the CGs with recommendations (61.6%).

‘New’ recommendations were mainly ‘new-added’ (294, 79.9%), from ‘gynaecology, pregnancy and birth’ topic (115, 31.3%), had a ‘treatment’ purpose (146, 39.7%), and were graded as ‘weak’ (308, 83.7%). The main reasons for the changes in the ‘new-replaced’ were the identification of new evidence (21/33; 63.6%) and changes in writing style (11/33; 33.3%) (Additional file 3).

### Presentation formats and information recording

The presentation formats used to indicate the changes in recommendations or sections varied across updated CGs. Five (5/9, 55.6%) updated CGs used update status labels for recommendations plus highlight colour [25,26,28,30,31]; three (3/9; 33.3%) used update status labels for recommendations plus a bar down the side of the page [15,29,14]; and one (1/9; 11.1%) only indicated the recommendations’ update status [27]. Updating status labels were defined in seven CGs (77.8%) [14,15,25,27,29-31] (definitions are available in Additional file 4).

The updated CGs with highest recording information score were for those that included a head to head comparison between original and updated recommendations in an appendix (10 points for both Caesarean section CG – 2011 [29] and Fertility CG – 2013 [14]); and the lowest scores were for the oldest updates (1.4 points for Head injury CG – 2007 [25] and 2.9 points for Chronic heart failure CG - 2010 [27]). An illustration of presentation formats is available in Additional file 5 and scores are available in Additional file 6.

Documented changes in recommendations were reported frequently in updated CGs appendices (n = 7; 77.8%) (Additional file 4). The changes in ‘amended’, ‘deleted’ and ‘new-replaced’ recommendations (n = 296) were recorded in 167 (56.4%) recommendations; and were explained in 81 (27.4%) recommendations. The most common change factor in ‘amended’ recommendations was ‘change in writing style (accuracy, clarity, consistency, or terminology)’ (n = 23, 65.7%), in ‘deleted’ recommendations was ‘recommendation outside the scope’ (n = 4; 30.8%); and in ‘new-replaced’ recommendations was ‘incorporation of new evidence’ (n = 21; 63.6%) (Additional file 3).

### Predictive factors for updating

After the linking process, we identified in original CGs: 229 (20.7%) ‘reviewed’ (‘deleted’ plus ‘new-replaced’ plus ‘not changed’), 783 (70.8%) ‘not reviewed’ and 94 (8.5)‘amended’ recommendations [Table 3]. A total of 7.1% (n = 78) had been ‘new-replaced’ (cases) and 2.4% (n = 27) were ‘not changed’ (controls) recommendations. More than one updated recommendation could correspond to one original recommendation and vice versa. For this reason, the absolute overall numbers of update status do not match between versions (Table 2).

There were differences between ‘new-replaced’ (cases) versus ‘not changed’ (controls) recommendations by CGs, topic and purpose, but not by time between publication versions or strength of recommendations (Table 4).

**Table 4:** Differences between recommendations by change status

	**New-replaced recommendations**		**Not changed recommendations**		**P**^**a**^
	**(cases, n = 78)**		**(controls, n = 27)**		
**CGs, n (%)**					
- Anaemia management in chronic kidney disease	1	(1.3)	-	-	0.036
- Caesarean section	10	(12.8)	1	(3.7)	
- Chronic heart failure	4	(5.1)	4	(14.8)	
- Chronic obstructive pulmonary disease	-	-	-	-	
- Epilepsy	2	(2.6)	5	(18.5)	
- Fertility	34	(43.6)	10	(37.0)	
- Head injury	3	(3.8)	-	-	
- Infection control	24	(30.8)	7	(25.9)	
- Lung cancer	-	-	-	-	
**Time between publication versions, median (range)**					
- Years	8.8	(4.3-9.0)	8.8	(7.1-9.0)	0.296
**CGs topic, n (%)**					
- Blood and immune system/Urogenital	1	(1.3)	-	-	0.027
- Cardiovascular	4	(5.1)	4	(14.8)	
- Central nervous system	2	(2.6)	5	(18.5)	
- Gynaecology, pregnancy and birth	44	(56.4)	11	(40.7)	
- Injuries, accidents and wounds	3	(3.8)	-	-	
- Public health	24	(30.8)	7	(25.9)	
- Respiratory	-	-	-	-	
**Recommendations purpose, n (%)**					
- Access to services - Referral and approach to care - Service organisation	3	(3.8)	2	(7.4)	0.027
- Diagnostic	7	(9.0)	1	(3.7)	
- Monitoring - Follow up	1	(1.3)	5	(18.5)	
- Prevention practices	23	(29.5)	7	(25.9)	
- Supporting patients and carers	17	(21.8)	2	(7.4)	
- Treatment	23	(29.5)	9	(33.3)	
- >1 procedure - Others	4	(5.1)	1	(3.7)	
**Recommendations strength - SIGN, n (%)**					
- A	25	(32.1)	6	(22.2)	0.296
- B	5	(6.4)	2	(7.4)	
- C	11	(14.1)	5	(18.5)	
- D	19	(24.4)	7	(25.9)	
- GPP	16	(20.5)	6	(22.2)	
- Others	2	(2.6)	1	(3.7)	

*Abbreviations: CG* Clinical guidelines, *GPP* Good practice point.

^a^Pearson Chi-Square or Mann-Whitney Test, as appropriate.

The ‘new-replaced’ recommendations (cases) were mainly from ‘Fertility (CG11) 2004′ CG [13], about ‘gynaecology, pregnancy and birth’ topic, and ‘treatment’ or ‘prevention’ purposes [Table 4].

We considered the sample of 105 recommendations inadequate to perform a multiple regression analysis [16].

## Discussion

Our study evaluated a cohort of updated recommendations in one of the leading national CG development programs. We identified 368 (28.1%) ‘new’ recommendations in the updated CGs; of these, 7.1% (78/1106) being ‘new-replaced’ recommendations from the original CGs. The changes were mainly due to the identification of new evidence and were topic and purpose related.

We included in the study partially updated guidelines; defined as CGs that include some recommendations that required updating in the light of new evidence, or because they were unclear [4]. The reason to include only partial updates is that in full updates there is no information about the modifications made with respect to the previous versions. Inclusion of only partial updates resulted in the majority of original recommendations not being reviewed. In some instances updated CGs can have the same original scope (no new areas were included), or in others a new scope is reported (where new key areas were identified) [4]. We did not assess the differences in scopes or included clinical questions between guidelines versions. It would have been useful for the analysis that all updated CGs recorded these issues. Only a minority of updated CGs included appendices highlighting how recommendations had changed between versions of the CG. Similarly there was no information reported about the methods undertaken for the review, surveillance or monitoring process.

The identification of the updated CGs was sometimes difficult because occasionally two or more CGs were merged, titles were modified, and some guidelines had went through more than one updating process. The expected increase of guideline updates in the future makes it necessary to improve labelling to avoid confusion. To further complicate the analysis, access to the original version of the CG was not straightforward, as they had been removed from the website. Nevertheless, we managed in all cases to obtain the original CGs as copies of the original guidelines are stored internally within NICE.

There was not a prioritisation for updating particular sections of the included CGs, hence, the distribution of reviewed recommendations was from across all guidelines and sections.

The proportion of 28.1% (368) ‘new’ recommendation over a median time frame of seven years since their development is similar to a recent survival analysis of the validity of recommendations (22.1 % recommendations were considered to need a potential update, median time frame of four years) [32]. A standardised process for conducting surveillance reviews every three years was introduced in August 2010 therefore; the median time frame of seven years between versions of CGs included in this study may be attributed partly because these CGs were reviewed for update on an *ad hoc* basis.

We observed unclear definition about update status labels, variability in the presentation formats for recommendation changes and poor reporting for the justification for change. Only two CGs included an appendix with a detailed comparison of the original and updated recommendations [14,29]. There is scant research in these areas with most focusing either on the presentation of original recommendations or in the field of systematic reviews [33-35].

An important area to highlight is the coexistence of two systems of strength of recommendations in the same updated CG. This is due to the relatively recent decision to move from the SIGN system to GRADE to rate the quality of the evidence and formulate the recommendations. At the moment, the impact of this potential confusion for users is unknown.

### Strengths and limitations

Our study has several strengths. First, we followed a structured and rigorous approach and developed a protocol that is available from the authors. Second, we explored all CGs from a national program that has a well-established reviewing and updating process. Finally, we explored several issues that have not been assessed until now (*e.g.*, updating reporting and presentation formats and predictive factors for updating recommendations).

Our study also has some limitations. We were not able to link recommendations with references supporting them, as originally planned. This could have led us to explore the association between number of references linked per recommendation and its vulnerability to change [35]. Furthermore, the identification of individual recommendations was difficult because, for example, more than one updated recommendation could correspond to an original recommendation, and vice versa. We might have therefore underestimated the real proportion of recommendations that were changed. Although the results suggest there were differences between changed versus not changed recommendations by CGs, topic and purpose, it was not possible to define predictive factors for updating recommendations. Additional work is needed to define changes to CG recommendations because this will help to focus evidence surveillance systems and, consequently, will improve the distribution of resources in the CG updating process. Finally, we only included the first update of each guideline and excluded later versions if available.

### Implications of our results for practice and research

Our study is the first to describe and assess updated CGs and recommendations from a national guideline program. Our results highlight the pressing need to standardise the reporting and presentation of updated recommendations. We have also identified a research gap about the optimal way to present updates to the users. Furthermore, there is a need for larger studies to investigate updating predictive factors.

### Availability of supporting data

The dataset is available from the corresponding author.

## Abbreviations

CG: Clinical guideline; GRADE: Grading of recommendations assessment, development and evaluation; NICE: National institute for health and care excellence; SIGN: Scottish intercollegiate guidelines network.

## Competing interests

The authors declare that they have no competing interests.

## Authors’ contributions

LMG (Laura Martínez García) and PAC (Pablo Alonso-Coello) participated in the conception of the study. LMG, EM (Emma McFarlane), SB (Steven Barnes) and PA (Philip Alderson) designed the study protocol. LMG carried out the data extraction and analysis and EM checked the coding and the link processes. LMG and EM wrote the first draft of the paper. All authors revised the article for important intellectual content and approved the final version of the submitted manuscript.

## Supplementary Material

Additional file 1 **Information about recoding.** We listed the key words used for recoding purpose, SIGN or GRADE system or update status recommendations. Click here for file

Additional file 2 **Sample selection.** We listed included and excluded CGs from NICE. Click here for file

Additional file 3 **Documented changes in recommendations.** We listed the documented changes by type of recommendation (amended, deleted or new). Click here for file

Additional file 4 **Presentation formats.** We listed the update status definition and the how and where the change were recorded and/or explained by CGs. Click here for file

Additional file 5 **Presentation formats examples.** We captured images form included updated clinical guidelines to illustrate different presentation formats. Click here for file

Additional file 6 **Reporting information score.** We listed the score by CGs. Click here for file
