# Supplementary material for: Updated recommendations: an assessment of NICE clinical guidelines
Source: Implement Sci. 2014 Jun 11;9:72. doi: 10.1186/1748-5908-9-72 (PMC4067507; doi:10.1186/1748-5908-9-72)
Supplement: Additional file 1 — Information about recoding. We listed the key words used for recoding purpose, SIGN or GRADE system or update status recommendations. [file 1748-5908-9-72-S1.pdf]

Additional File 1: Information about recoding (purpose)

Access to services - Referral and approach to care - Service organisation [Access]	Diagnostic	Monitoring - Follow up [Monitoring]	Prevention practices [Prevention]	Supporting patients and carers [Supporting]	Treatment	>1 procedure - Others [Others]
access to	analysis	follow	alcohol	advice	resp	
admission	assess	measured	catheter	agree	administration	
admission	CT	monitor	clean	aware	dosing	
available	define	observations	container	card	drug	
care	diagnos	reappraisal	contaminat	carer	effecti	
centre	EEG	review	drainage	communication	indicat	
clinician	examination	others	dressing	counselling	intervention	
consultant	exclude		exercise	discuss	manag	
discharg	identif		full-body fluid-repellen	educat	medication	
document	imaging		gloves	empower	pharma	
environment	investigation		hygiene	encourag	prescribe	
guideline	measure		infect	enquiry	prescription	
hospital	MRI		lifestyle	famil	programmes	
multidisciplinary	radiograph		needle	inform	surg	
network	scan		no-touch technique	instruction	surgery	
nurse	spirometry		plastic aprons	participate	therap	
protocol	stud		prevent	preference	treat	
refer	suspect		prophyla	relative	others	
service	test		protect	self		
setting	X-ray		ready-to-use feeds	share		
specialist	others		risk	support		
staff			screening	others		
team			septic			
transfer			sharp			
unit			single (use)			
others			smoke			
			sterile			
			vaccin			

Additional File 1: Information about recoding (purpose)

			wash			
			others			

Additional File 1: Information about recoding (SIGN)

A	B	C	D	GPP	Others
A	B	C	D	GPP	>1
A (DS)	B (DS)	C (DS)	D (GPP)		HSC
A (NICE)			D H&S		NICE
					H&S

Abbreviations: DS: Diagnostic Studies; GPP: Good Practice Point; H&S: Health and Safety (H&S) requirement; HSC: Evidence from Health Service Circulars; NICE: Evidence from NICE guidelines or Health Technology

Additional File 1: Information about recoding (GRADE)

Legal	Strong	Weak
Must	Should	Consider
Must and Should	Ensure	*[Without others]*
	Should and Consider	

Additional File 1: Information about recoding (update status)

Amended	New	Not changed	Not reviewed
[2003, amended 2010]	[2007]	[2010]	-
[2003, amended 2010] KPI	[new 2010]	[2011]	[2003]
[2003, amended 2012]	[new 2010] KPI	[2012]	[2003] KPI
[2004, amended 2011]	[new 2011]	[2013]	[2004]
[2004, amended 2012]	[new 2012]	Not changing	[2005]
[2004, amended 2013]	[new 2013]		[without orange color]
[2006, amended 2011]	[NEW]		[without pink color]
[Amended]	New		
[partially highlighted in orange	NEW 2010		
[partially highlighted in pink color]			
