# Supplementary material for: Updated recommendations: an assessment of NICE clinical guidelines
Source: Implement Sci. 2014 Jun 11;9:72. doi: 10.1186/1748-5908-9-72 (PMC4067507; doi:10.1186/1748-5908-9-72)
Supplement: Additional file 2 — Sample selection. We listed included and excluded CGs from NICE. [file 1748-5908-9-72-S2.pdf]

Additional File 2: Sample selection

Id	Ref	CGs	Date Issued	Review	Version	Selection	Exclusion reason
**1**	C	Electronic fetal monitoring (C) (replaced by CG55)	may-01		OriginalCG, replaced	Excluded	
**2**	D	Induction of labour (D) (replaced by CG70)	jun-01		OriginalCG, replaced	Excluded	
**3**	A	Myocardial infarction (A) (replaced by CG48) (withdrawn)	abr-01		OriginalCG, replaced	Excluded	
**4**	B	Pressure ulcers (see Pressure ulcer management, CG29) (B)	abr-01		OriginalCG, reviewed but not updated		
**5**	G	Type 2 diabetes - blood glucose (G) (replaced by CG66)	sep-02		OriginalCG, replaced	Excluded	
**6**	H	Type 2 diabetes - management of blood pressure and blood lipids (H) (replaced by CG66)	oct-02		OriginalCG, replaced	Excluded	
**7**	F	Type 2 diabetes - renal disease (F) (replaced by CG66)	feb-02		OriginalCG, replaced	Excluded	
**8**	E	Type 2 diabetes - retinopathy (E) (replaced by CG66)	feb-02		OriginalCG, replaced	Excluded	
**9**	CG1	Schizophrenia (CG1) (replaced by CG82)	dic-02		OriginalCG, replaced	Excluded	
**10**	CG2	Infection control (CG2) (replaced by CG139)	jun-03		OriginalCG, replaced	Included	
**11**	CG3	Preoperative tests (CG3)	jun-03		OriginalCG, not reviewed		
**12**	CG4	Head injury (CG4) (replaced by CG56) (withdrawn)	jun-03		OriginalCG, replaced	Included	
**13**	CG5	Chronic heart failure (CG5) (replaced by CG108)	jul-03		OriginalCG, replaced	Included	
**14**	CG6	Antenatal care (CG6) (replaced by CG62)	oct-03		OriginalCG, replaced	Excluded	
**15**	CG7	Pressure relieving devices (CG7)	oct-03	may-11	OriginalCG, reviewed but not updated		
**16**	CG8	Multiple sclerosis (CG8)	nov-03	jun-11	OriginalCG, reviewed but not updated		
**17**	CG9	Eating disorders (CG9)	ene-04		OriginalCG, not reviewed		
**18**	CG10	Type 2 diabetes - footcare (CG10)	ene-04	ago-11	OriginalCG, reviewed but not updated		
**19**	CG11	Fertility (CG11) (replaced by CG156)	feb-04		OriginalCG, replaced	Included	
**20**	CG12	Chronic obstructive pulmonary disease (CG12) (replaced by CG101)	feb-04		OriginalCG, replaced	Included	
**21**	CG13	Caesarean section (replaced by CG132) (CG13)	abr-04		OriginalCG, replaced	Included	

Additional File 2: Sample selection

**22**	CG14	Familial breast cancer (CG14) (replaced by CG41) (withdrawn)	may-04		OriginalCG, replaced	Excluded	
**23**	CG15	Type 1 diabetes (CG15)	jul-04	ago-11	OriginalCG, reviewed but not updated		
**24**	CG16	Self-harm (CG16)	jul-04	feb-12	OriginalCG, reviewed but not updated		
**25**	CG17	Dyspepsia (CG17)	ago-04	jul-11	OriginalCG, reviewed but not updated		
**26**	CG18	Hypertension (CG18) (replaced by CG34) (withdrawn)	ago-04		OriginalCG, replaced	Excluded	
**27**	CG19	Dental recall (CG19)	oct-04	ago-12	OriginalCG, reviewed but not updated		
**28**	CG20	Epilepsy (CG20) (replaced by CG137)	oct-04		OriginalCG, replaced	Included	
**29**	CG21	Falls (CG21)	nov-04	jul-11	OriginalCG, reviewed but not updated		
**30**	CG22	Anxiety (CG22) (replaced by CG113)	dic-04		OriginalCG, replaced	Excluded	
**31**	CG23	Depression (CG23) (replaced by CG90)	dic-04		OriginalCG, replaced	Excluded	
**32**	CG24	Lung cancer (CG24) (replaced by CG121)	feb-05		OriginalCG, replaced	Included	
**33**	CG25	Violence (CG25)	feb-05	feb-12	OriginalCG, reviewed but not updated		
**34**	CG26	Post-traumatic stress disorder (PTSD) (CG26)	mar-05		OriginalCG, not reviewed		
**35**	CG27	Referral for suspected cancer (CG27)	jun-05		OriginalCG, not reviewed		
**36**	CG28	Depression in children and young people (CG28)	sep-05	feb-11	OriginalCG, reviewed but not updated		
**37**	CG29	Pressure ulcer management (CG29)	sep-05	may-11	OriginalCG, reviewed but not updated		
**38**	CG30	Long-acting reversible contraception (CG30)	oct-05	mar-11	OriginalCG, reviewed but not updated		
**39**	CG31	Obsessive compulsive disorder (OCD) and body dysmorphic disorder (BDD) (CG31)	nov-05		OriginalCG, not reviewed		
**40**	CG32	Nutrition support in adults (CG32)	feb-06	jun-11	OriginalCG, reviewed but not updated		
**41**	CG33	Tuberculosis (CG33) (replaced by CG117)	mar-06		OriginalCG, replaced	Excluded	
**42**	CG34	Hypertension (CG34) (replaced by CG127)	jun-06		UpdatedCG (first update)	Excluded	Not update status
**43**	CG35	Parkinson's disease (CG35)	jun-06	jul-11	OriginalCG, reviewed but not updated		
**44**	CG36	Atrial fibrillation (CG36)	jun-06	ago-11	OriginalCG, reviewed but not updated		
**45**	CG37	Postnatal care (CG37)	jul-06	feb-12	OriginalCG, reviewed but not updated		
**46**	CG38	Bipolar disorder (CG38)	jul-06	jul-11	OriginalCG, reviewed but not updated		
**47**	CG39	Anaemia management in chronic kidney disease (CG39) (replaced by CG114)	sep-06		OriginalCG, replaced	Included	
**48**	CG40	Urinary incontinence (CG40)	oct-06		OriginalCG, not reviewed		
**49**	CG41	Familial breast cancer (CG41)	oct-06		UpdatedCG (first update)	Excluded	Not partial update
**50**	CG42	Dementia (CG42)	nov-06	abr-12	OriginalCG, reviewed but not updated		

Additional File 2: Sample selection

**51**	CG43	Obesity (CG43)	dic-06	dic-11	OriginalCG, reviewed but not updated		
**52**	CG44	Heavy menstrual bleeding (CG44)	ene-07	ene-12	OriginalCG, reviewed but not updated		
**53**	CG45	Antenatal and postnatal mental health (CG45)	feb-07	jul-11	OriginalCG, reviewed but not updated		
**54**	CG46	Venous thromboembolism (surgical) (CG46) (replaced by CG92) (withdrawn)	abr-07		OriginalCG, replaced	Excluded	
**55**	CG47	Feverish illness in children (CG47)	may-07	ene-11	OriginalCG, reviewed but not updated		
**56**	CG48	MI: secondary prevention (CG48)	may-07	feb-11	UpdatedCG (first update)	Excluded	Not partial update
**57**	CG49	Faecal incontinence (CG49)	jun-07	dic-10	OriginalCG, reviewed but not updated		
**58**	CG50	Acutely ill patients in hospital (CG50)	jul-07	dic-10	OriginalCG, reviewed but not updated		
**59**	CG51	Drug misuse: psychosocial interventions (CG51)	jul-07	mar-11	OriginalCG, reviewed but not updated		
**60**	CG52	Drug misuse: opioid detoxification (CG52)	jul-07	mar-11	OriginalCG, reviewed but not updated		
**61**	CG53	Chronic fatigue syndrome / Myalgic encephalomyelitis (CG53)	ago-07	mar-11	OriginalCG, reviewed but not updated		
**62**	CG54	Urinary tract infection in children (CG54)	ago-07	may-11	OriginalCG, reviewed but not updated		
**63**	CG55	Intrapartum care (CG55)	sep-07		UpdatedCG (first update)	Excluded	Not partial update
**64**	CG56	Head injury (CG56)	sep-07	mar-11	UpdatedCG (first update)	Included	
**65**	CG57	Atopic eczema in children (CG57)	dic-07		OriginalCG, not reviewed		
**66**	CG58	Prostate cancer (CG58)	feb-08	jul-11	OriginalCG, reviewed but not updated		
**67**	CG59	Osteoarthritis (CG59)	feb-08	jun-11	OriginalCG, reviewed but not updated		
**68**	CG60	Surgical management of OME (CG60)	feb-08		OriginalCG, not reviewed		
**69**	CG61	Irritable bowel syndrome (CG61)	feb-08		OriginalCG, not reviewed		
**70**	CG62	Antenatal care (CG62)	mar-08	may-11	UpdatedCG (first update)	Excluded	Not update status
**71**	CG63	Diabetes in pregnancy (CG63)	mar-08	may-11	OriginalCG, reviewed but not updated		
**72**	CG64	Prophylaxis against infective endocarditis (CG64)	mar-08		OriginalCG, not reviewed		
**73**	CG65	Perioperative hypothermia (inadvertent) (CG65)	abr-08	nov-11	OriginalCG, reviewed but not updated		
**74**	CG66	Type 2 diabetes (partially updated by CG87) (CG66)	may-08	ago-11	UpdatedCG (first update)	Excluded	Not partial update
**75**	CG67	Lipid modification (CG67)	may-08		OriginalCG, not reviewed		
**76**	CG68	Stroke (CG68)	jul-08	abr-12	OriginalCG, reviewed but not updated		
**77**	CG69	Respiratory tract infections (CG69)	jul-08	jun-12	OriginalCG, reviewed but not updated		
**78**	CG70	Induction of labour (CG70)	jul-08	ago-11	UpdatedCG (first update)	Excluded	Not partial update
**79**	CG71	Familial hypercholesterolaemia (CG71)	ago-08	ago-11	OriginalCG, reviewed but not updated		

Additional File 2: Sample selection

**80**	CG72	Attention deficit hyperactivity disorder (ADHD) (CG72)	sep-08	nov-11	OriginalCG, reviewed but not updated		
**81**	CG73	Chronic kidney disease (CG73)	sep-08	dic-11	OriginalCG, reviewed but not updated		
**82**	CG74	Surgical site infection (CG74)	oct-08		OriginalCG, not reviewed		
**83**	CG75	Metastatic spinal cord compression (CG75)	nov-08	ago-12	OriginalCG, reviewed but not updated		
**84**	CG76	Medicines adherence (CG76)	ene-09		OriginalCG, not reviewed		
**85**	CG77	Antisocial personality disorder (CG77)	ene-09	ene-12	OriginalCG, reviewed but not updated		
**86**	CG78	Borderline personality disorder (BPD) (CG78)	ene-09	ene-12	OriginalCG, reviewed but not updated		
**87**	CG79	Rheumatoid arthritis (CG79)	feb-09	nov-11	OriginalCG, reviewed but not updated		
**88**	CG80	Breast cancer (early & locally advanced) (CG80)	feb-09	mar-12	OriginalCG, reviewed but not updated		
**89**	CG81	Breast cancer (advanced) (CG81)	feb-09	mar-12	OriginalCG, reviewed but not updated		
**90**	CG82	Schizophrenia (update) (CG82)	mar-09	ago-11	UpdatedCG (first update)	Excluded	Not partial update
**91**	CG83	Critical illness rehabilitation (CG83)	mar-09	jun-12	OriginalCG, reviewed but not updated		
**92**	CG84	Diarrhoea and vomiting in children under 5 (CG84)	abr-09	jul-12	OriginalCG, reviewed but not updated		
**93**	CG85	Glaucoma (CG85)	abr-09	ago-12	OriginalCG, reviewed but not updated		
**94**	CG86	Coeliac disease (CG86)	may-09	jul-12	OriginalCG, reviewed but not updated		
**95**	CG87	Type 2 Diabetes - newer agents (partial update of CG66) (CG87)	may-09	ago-11	UpdatedCG (second update)		
**96**	CG88	Low back pain (CG88)	may-09	jun-12	OriginalCG, reviewed but not updated		
**97**	CG89	When to suspect child maltreatment (CG89)	jul-09	ago-12	OriginalCG, reviewed but not updated		
**98**	CG90	Depression in adults (update) (CG90)	oct-09	oct-12	UpdatedCG (first update)	Excluded	Not partial update
**99**	CG91	Depression with a chronic physical health problem (CG91)	oct-09	oct-12	OriginalCG, reviewed but not updated		
**100**	CG92	Venous thromboembolism - reducing the risk (CG92)	ene-10	ene-13	UpdatedCG (first update)	Excluded	Not partial update
**101**	CG93	Donor breast milk banks (CG93)	feb-10	feb-13	OriginalCG, not reviewed		
**102**	CG94	Unstable angina and NSTEMI (CG94)	mar-10	mar-13	OriginalCG, not reviewed		
**103**	CG95	Chest pain of recent onset (CG95)	mar-10	mar-13	OriginalCG, not reviewed		
**104**	CG96	Neuropathic pain - pharmacological management (CG96)	mar-10		OriginalCG, not reviewed		
**105**	CG97	Lower urinary tract symptoms (CG97)	may-10	may-13	OriginalCG, not reviewed		
**106**	CG98	Neonatal jaundice (CG98)	may-10	may-13	OriginalCG, not reviewed		

Additional File 2: Sample selection

**107**	CG99	Constipation in children and young people (CG99)	may-10	may-13	OriginalCG, not reviewed		
**108**	CG100	Alcohol-use disorders: physical complications (CG100)	jun-10	jun-13	OriginalCG, not reviewed		
**109**	CG101	Chronic obstructive pulmonary disease (updated) (CG101)	jun-10	jun-13	UpdatedCG (first update)	Included	
**110**	CG102	Bacterial meningitis and meningococcal septicaemia (CG102)	jun-10	jun-13	OriginalCG, not reviewed		
**111**	CG103	Delirium (CG103)	jul-10	jul-13	OriginalCG, not reviewed		
**112**	CG104	Metastatic malignant disease of unknown primary origin (CG104)	jul-10	jul-13	OriginalCG, not reviewed		
**113**	CG105	Motor neurone disease - non-invasive ventilation (CG105)	jul-10	jul-13	OriginalCG, not reviewed		
**114**	CG106	Barrett's oesophagus - ablative therapy (CG106)	ago-10	ago-13	OriginalCG, not reviewed		
**115**	CG107	Hypertension in pregnancy (CG107)	ago-10	ago-13	OriginalCG, not reviewed		
**116**	CG108	Chronic heart failure (CG108)	ago-10	ago-13	UpdatedCG (first update)	Included	
**117**	CG109	Transient loss of consciousness in adults and young people (CG109)	ago-10	ago-13	OriginalCG, not reviewed		
**118**	CG110	Pregnancy and complex social factors (CG110)	sep-10	sep-13	OriginalCG, not reviewed		
**119**	CG111	Nocturnal enuresis - the management of bedwetting in children and young people (CG111)	oct-10	oct-13	OriginalCG, not reviewed		
**120**	CG112	Sedation in children and young people (CG112)	dic-10		OriginalCG, not reviewed		
**121**	CG113	Anxiety (CG113)	ene-11		UpdatedCG (first update)	Excluded	Not update status
**122**	CG114	Anaemia management in people with chronic kidney disease (CG114)	feb-11	dic-11	UpdatedCG (first update)	Included	
**123**	CG115	Alcohol dependence and harmful alcohol use (CG115)	feb-11		OriginalCG, not reviewed		
**124**	CG116	Food allergy in children and young people (CG116)	feb-11		OriginalCG, not reviewed		
**125**	CG117	Tuberculosis (CG117)	mar-11		UpdatedCG (first update)	Excluded	Not update status

Additional File 2: Sample selection

**126**	CG118	Colonoscopic surveillance for prevention of colorectal cancer in people with ulcerative colitis, Crohn's disease or adenomas (CG118)	mar-11		OriginalCG, not reviewed		
**127**	CG119	Diabetic foot problems - inpatient management (CG119)	mar-11		OriginalCG, not reviewed		
**128**	CG120	Psychosis with coexisting substance misuse (CG120)	mar-11		OriginalCG, not reviewed		
**129**	CG121	Lung cancer (CG121)	abr-11		UpdatedCG (first update)	Included	
**130**	CG122	Ovarian cancer (CG122)	abr-11		OriginalCG, not reviewed		
**131**	CG123	Common mental health disorders (CG123)	may-11		OriginalCG, not reviewed		
**132**	CG124	Hip fracture (CG124)	jun-11	TBC	OriginalCG, not reviewed		
**133**	CG125	Peritoneal dialysis (CG125)	jul-11		OriginalCG, not reviewed		
**134**	CG126	Stable angina (CG126)	jul-11		OriginalCG, not reviewed		
**135**	CG127	Hypertension (CG127)	ago-11		UpdatedCG (second update)		
**136**	CG128	Autism in children and young people (CG128)	sep-11		OriginalCG, not reviewed		
**137**	CG129	Multiple pregnancy (CG129)	sep-11		OriginalCG, not reviewed		
**138**	CG130	Hyperglycaemia in acute coronary syndromes (CG130)	oct-11		OriginalCG, not reviewed		
**139**	CG131	Colorectal cancer (CG131)	nov-11		OriginalCG, not reviewed		
**140**	CG132	Caesarean section (CG132)	nov-11		UpdatedCG (first update)	Included	
**141**	CG133	Self-harm (longer term management) (CG133)	nov-11		OriginalCG, not reviewed		
**142**	CG134	Anaphylaxis (CG134)	dic-11		OriginalCG, not reviewed		
**143**	CG135	Organ donation (CG135)	dic-11		OriginalCG, not reviewed		
**144**	CG136	Service user experience in adult mental health (CG136)	dic-11		OriginalCG, not reviewed		
**145**	CG137	Epilepsy (CG137)	ene-12		UpdatedCG (first update)	Included	
**146**	CG138	Patient experience in adult NHS services (CG138)	feb-12		OriginalCG, not reviewed		
**147**	CG139	Infection control (CG139)	mar-12		UpdatedCG (first update)	Included	
**148**	CG140	Opioids in palliative care (CG140)	may-12		OriginalCG, not reviewed		
**149**	CG141	Acute upper GI bleeding (CG141)	jun-12		OriginalCG, not reviewed		
**150**	CG142	Autism in adults (CG142)	jun-12		OriginalCG, not reviewed		
**151**	CG143	Sickle cell acute painful episode (CG143)	jun-12		OriginalCG, not reviewed		

Additional File 2: Sample selection

**152**	CG144	Venous thromboembolic diseases (CG144)	jun-12		OriginalCG, not reviewed		
**153**	CG145	Spasticity in children and young people (CG145)	jul-12		OriginalCG, not reviewed		
**154**	CG146	Osteoporosis fragility fracture (CG146)	ago-12		OriginalCG, not reviewed		
**155**	CG147	Lower limb peripheral arterial disease (CG147)	ago-12		OriginalCG, not reviewed		
**156**	CG148	Urinary incontinence in neurological disease (CG148)	ago-12		OriginalCG, not reviewed		
**157**	CG149	Antibiotics for early-onset neonatal infection (CG149)	ago-12		OriginalCG, not reviewed		
**158**	CG150	Headaches (CG150)	sep-12		OriginalCG, not reviewed		
**159**	CG151	Neutropenic sepsis (CG151)	sep-12		OriginalCG, not reviewed		
**160**	CG152	Crohn's disease (CG152)	oct-12		OriginalCG, not reviewed		
**161**	CG153	Psoriasis (CG153)	oct-12		OriginalCG, not reviewed		
**162**	CG154	Ectopic pregnancy and miscarriage (CG154)	dic-12		OriginalCG, not reviewed		
**163**	CG155	Psychosis and schizophrenia in children and young people (CG155)	ene-13		OriginalCG, not reviewed		
**164**	CG156	Fertility (CG156)	feb-13		UpdatedCG (first update)	Included	
**165**	CG157	Hyperphosphataemia in chronic kidney disease (CG157)	mar-13		OriginalCG, not reviewed		
**166**	CG158	Conduct disorders in children and young people (CG158)	mar-13		OriginalCG, not reviewed		

Source: http://guidance.nice.org.uk/CG/Published (this page was last updated: 09 May 2013)
Accessed : 15/05/2013
