# Supplementary material for: Updated recommendations: an assessment of NICE clinical guidelines
Source: Implement Sci. 2014 Jun 11;9:72. doi: 10.1186/1748-5908-9-72 (PMC4067507; doi:10.1186/1748-5908-9-72)
Supplement: Additional file 3 — Documented changes in recommendations. We listed the documented changes by type of recommendation (amended, deleted or new). [file 1748-5908-9-72-S3.pdf]

Additional File 3: Documented changes in recommendations

	Amended recommendations (n=35)		Deleted recommendations (n=13)		New-replaced recommendations (n=33)	
**Documented changes***	n	%	n	%	n	%
. Conflicted with others recommendations	-	-	2	15,4	-	-
. Drug licence	2	5,7	-	-	-	-
. New evidence	1	2,9	2	15,4	21	63,6
. New scope	3	8,6	-	-	-	-
. Recommendation not applicable	-	-	3	23,1	-	-
. Outside the scope	-	-	4	30,8	-	-
. Population	1	2,9	-	-	2	6,1
. Reference guideline updated	4	11,4	-	-	-	-
. Writing style (accuracy, clarity, consistency, terminology)	23	65,7	-	-	11	33,3
. Others	2	5,7	2	15,4	2	6,1
*More than one possible category						
