# Supplementary material for: Updated recommendations: an assessment of NICE clinical guidelines
Source: Implement Sci. 2014 Jun 11;9:72. doi: 10.1186/1748-5908-9-72 (PMC4067507; doi:10.1186/1748-5908-9-72)
Supplement: Additional file 4 — Presentation formats. We listed the update status definition and the how and where the change were recorded and/or explained by CGs. [file 1748-5908-9-72-S4.pdf]

Additional File 4: Presentation formats

Id	CGs	Update status labels?	Highlight colour?	Bar down the side of the page?	Update status definition	Change recorded and/or explained
1	Anaemia management in people with chronic kidney disease (CG114), 2011	Yes	Yes	No	Our assumption:<br>• **without orange color** if the evidence has not been reviewed since the original guideline.<br>• **partially highlighted in orange color or [2006, amended 2011]** if the evidence has not been reviewed, but an essential change has been made that affects the meaning of the recommendation.<br>• **[2011]** if the evidence has been reviewed but no change has been made to the recommendation.<br>• **[new 2011]** if the evidence has been reviewed and the recommendation has been updated or added.	• **Amended** recommendations partially highlighted in orange color and with footnotes<br>• **Deleted** recommendations in "Appendix J: Deleted parts from the 2006 guideline (no longer relevant)" (pg. 544 full CG)
2	Caesarean section (CG132), 2011	Yes	No	Yes	Quote (pg. 2 full CG):<br>*"Recommendations are marked to indicate the year of the last evidence review:*<br>• ***[2004]*** *if the evidence has not been reviewed since the original guideline.*<br>• ***[2004], amended [2011]*** *if the evidence has not been reviewed, but an essential change has been made that affects the meaning of the recommendation.*<br>• ***[2011]*** *if the evidence has been reviewed but no change has been made to the recommendation.*<br>• ***[new 2011]*** *if the evidence has been reviewed and the recommendation has been updated or added."*	• **Amended, new and deleted** recommendations in "Appendix J Changes to original recommendations"
3	Chronic heart failure (CG108), 2010	Yes	No	No	Quote (pg. 32 full CG):<br>*"The status of each recommendation is indicated as follows:*<br>• ***[2003]*** *: Recommendation from the 2003 guideline where the evidence has not been formally reviewed for the 2010 update.*<br>• ***[2003, amended 2010]*** *: A small amendment has been made to the 2003 recommendation but the evidence has not been updated or reviewed.*<br>• ***[2010]*** *: Recommendation from the 2003 guideline where evidence has been reviewed but the recommendation is not changed. (This includes recommendations which are reworded in a new direct style.)*<br>• ***[new 2010]*** *: Recommendation from 2003 guideline which has been changed following review of evidence; or New recommendation following review of evidence"*	• **Deleted** recommendations in "Appendix N – 2003 deleted recommendations"

**4**	Chronic obstructive pulmonary disease (updated) (CG101), 2010	Yes	Yes	No	Our assumption:<br>• **without pink color** Recommendation from the 2004 guideline where the evidence has not been formally reviewed for the 2010 update.<br>• **partially highlighted in pink color** A small amendment has been made to the 2004 recommendation but the evidence has not been updated or reviewed.<br>• **[new 2010]** Recommendation from 2004 guideline which has been changed following review of evidence; or New recommendation following review of evidence<br>• **[Deleted]**	• **Amended** recommendations partially highlighted in pink color<br>• **Deleted** recommendations in "Appendix K NEW 2010 deleted sections from original guideline" (pg. 531 full CG)
**5**	Epilepsy (CG137), 2012	Yes	Yes	No	Quote (pg. 28 full CG):<br>*"Labelling of recommendations*<br>*• New recommendations are defined as either an additional area for the guideline or changed because of an updated evidence review. New recommendations are labelled by adding* ***[NEW 2012]*** *to the end of the recommendation.*<br>*• Unchanged recommendations where the evidence has been reviewed for the 2012 update are labelled as* ***[2012]****. These recommendations could be reworded to match new-style recommendations but the developers checked with the GDG that rewording hasn't changed the meaning.*<br>*• Unchanged recommendations from 2004, where the evidence has not been formally reviewed for the 2011 update, are labelled as* ***[2004]****.*<br>*• Where evidence has not been reviewed, but there have been minor changes in 2012 to the wording of a 2004 recommendation that do not affect the meaning, for specific reasons such as in terminology or availability of drugs, these are labelled as* ***[2004, amended 2012]****.*<br>*Deleted recommendations from the 2004 guideline can be viewed in Appendix X"*	• **Amended** recommendations with footnotes<br>• **Deleted** recommendations in "APPENDIX V Removed sections from original guideline"
**6**	Fertility (CG156), 2013	Yes	No	Yes	Quote (pg. 4 full CG):<br>*"Recommendations are marked to indicate the year and type of review:*<br>*•* ***[2004]*** *if the evidence has not been reviewed since the original guideline.*<br>*•* ***[2004, amended 2013]*** *if the evidence has not been reviewed, but an essential change has been made that affects the meaning of the recommendation.*<br>*•* ***[2013]*** *if the evidence has been reviewed but no change has been made to the recommendation.*<br>*•* ***[new 2013]*** *if the evidence has been reviewed and the recommendation has been updated or added."*	• **Amended, new and deleted** recommendations in "Appendix L Proposed changes to original recommendations"

**7**	Head injury (CG56), 2007	Yes	Yes	No	Quote (pg. 30 full CG):<br>*"In this update, there are new recommendations in the sections on prehospital management, emergency department assessment, investigations for clinically important brain injuries, investigation for non-accidental injury in children, and transfer from secondary settings. These are highlighted in the document as **'New'** . A number of amendments have been made to other recommendations from the initial guideline, and these are highlighted in the document as **'Amended'** ."*	-
**8**	Infection control (CG139), 2012	Yes	Yes	No	Quote (pg. 4 full CG):<br>*"Recommendations are marked to indicate the year of the last evidence review:*<br>*• **[2003]** if the evidence has not been updated since the original guideline,*<br>*• **[2003, amended 2012]** if the evidence has not been updated since the original guideline, but changes have been made that alter the meaning of the recommendation,*<br>*• **[2012]** if the evidence has been reviewed but no change has been made to the recommendation and*<br>*• **[new 2012]** if the evidence has been reviewed and the recommendation has been added or updated."*	• **Amended, new and deleted** recommendations in "Appendix D: D.10 Deleted and amended recommendations (2003)"
**9**	Lung cancer (CG121), 2011	Yes	No	Yes	Quote (pg. 1 full CG):<br>*"Recommendations are marked as [2005], [2011] or [new 2011].*<br>***[2005]** indicates that the evidence has not been updated and reviewed since 2005.*<br>***[2011]** indicates that the evidence has been reviewed but no changes have been made to the recommendation.*<br>***[new 2011]** indicates that the evidence has been reviewed and the recommendation has been added or updated."*	-
