# Supplementary material for: Updated recommendations: an assessment of NICE clinical guidelines
Source: Implement Sci. 2014 Jun 11;9:72. doi: 10.1186/1748-5908-9-72 (PMC4067507; doi:10.1186/1748-5908-9-72)
Supplement: Additional file 5 — Presentation formats examples. We captured images form included updated clinical guidelines to illustrate different presentation formats. [file 1748-5908-9-72-S5.pdf]

Additional file 3: Presentation format example - recommendations update status labels plus highlight colour

Caesarean section (CG132)

4.2.4.4 General principles for management of vascular access devices

88. Decontaminate the injection port or vascular access device catheter hub before and after accessing the system using chlorhexidine gluconate in 70% alcohol. Consider using an aqueous solution of chlorhexidine gluconate if the manufacturer's recommendations prohibit the use of alcohol with their catheter. [new 2012]

Update 2012

89. In-line filters should not be used routinely for infection prevention. [2003]

90. Antibiotic lock solutions should not be used routinely to prevent catheter-related bloodstream infections (CRBSI). [2003]

91. Systemic antimicrobial prophylaxis should not be used routinely to prevent catheter colonisation or CRBSI, either before insertion or during the use of a central venous catheter. [2003]

92. Preferably, a single lumen catheter should be used to administer parenteral nutrition. If a multilumen catheter is used, one port must be exclusively dedicated for total parenteral nutrition, and all lumens must be handled with the same meticulous attention to aseptic technique. [2003]

Reference: National Institute for Clinical Excellence. CG132 - Caesarean section (update). http://guidance.nice.org.uk/CG132 (accessed 15 May, 2013).

Additional file 3: Presentation format example - recommendations update status labels plus bar down the side of the page

Infection control (CG139)

Provision of information

1 Pregnant women should be offered evidence-based information and support to enable them to make informed decisions about childbirth. Addressing women's views and concerns should be recognised as being integral to the decision-making process. [C] **[2004]** 4.1

2 Give pregnant women evidence-based information about CS during the antenatal period, because about 1 in 4 women will have a CS. Include information about CS, such as: 4.1

- indications for CS (such as presumed fetal compromise, 'failure to progress' in labour, breech presentation)
- what the procedure involves
- associated risks and benefits
- implications for future pregnancies and birth after CS. [GPP] **[new 2011]**

2011 update

Reference: National Institute for Clinical Excellence. CG139 - Infection: prevention and control of healthcare-associated infections in primary and community care. http://guidance.nice.org.uk/CG139 (accessed 15 May, 2013).

Additional file 3: Presentation format example - head to head comparison between original and updated recommendations

Fertility (CG156)

Recommendation	Replaced with	Reason for change/deletion
	response and less than 4 IU/l for a high response[6]. **[new 2013]**	
Women should be informed that the value of assessing ovarian reserve using inhibin B is uncertain and is therefore not recommended. [C]	Do not use any of the following tests individually to predict any outcome of fertility treatment:<br>• ovarian volume<br>• ovarian blood flow<br>• inhibin B<br>• oestradiol (E2). **[new 2013]**	New evidence on ovarian reserve was reviewed and and the recommendation has been updated accordingly.

Reference: National Institute for Clinical Excellence. CG156 - Fertility: assessment and treatment for people with fertility problems. http://guidance.nice.org.uk/CG156 (accessed 15 May, 2013).
