# Supplementary material for: Updated recommendations: an assessment of NICE clinical guidelines
Source: Implement Sci. 2014 Jun 11;9:72. doi: 10.1186/1748-5908-9-72 (PMC4067507; doi:10.1186/1748-5908-9-72)
Supplement: Additional file 6 — Reporting information score. We listed the score by CGs. [file 1748-5908-9-72-S6.pdf]

**Additional file 6: Reporting information score**

| Id | CGs | Recommendation update status defined? | Amended - Changes recorded? | Amended - Changes explanation? | Deleted - Changes recorded? | Deleted - Changes explanation? | New (replaced) - Changes recorded? | New (replaced) - Changes explanation? | Points | Max possible points | 10 Score |
|---|---|---|---|---|---|---|---|---|---|---|---|
| 1 | Anaemia management in people with chronic kidney disease (CG114), 2011 | 0 | 1 | 1 | 1 | 0 | 0 | 0 | 3 | 7 | 4,3 |
| 2 | Caesarean section (CG132), 2011 | 1 | 1 | 1 | 1 | 1 | 1 | 1 | 7 | 7 | 10,0 |
| 3 | Chronic heart failure (CG108), 2010 | 1 | 0 | 0 | 1 | 0 | 0 | 0 | 2 | 7 | 2,9 |
| 4 | Chronic obstructive pulmonary disease (updated) (CG101), 2010 | 0 | 1 | 0 | 1 | 0 | - | - | 2 | 5 | 4,0 |
| 5 | Epilepsy (CG137), 2012 | 1 | 1 | 1 | 1 | 1 | 0 | 0 | 5 | 7 | 7,1 |
| 6 | Fertility (CG156), 2013 | 1 | 1 | 1 | 1 | 1 | 1 | 1 | 7 | 7 | 10,0 |
| 7 | Head injury (CG56), 2007 | 1 | 0 | 0 | 0 | 0 | 0 | 0 | 1 | 7 | 1,4 |
| 8 | Infection control (CG139), 2012 | 1 | 1 | 1 | 1 | 0 | 1 | 0 | 5 | 7 | 7,1 |
| 9 | Lung cancer (CG121), 2011 | 1 | - | - | 0 | 0 | - | - | 1 | 3 | 3,3 |
